# Effects of Deoxynivalenol and Fumonisins on Broiler Gut Cytoprotective Capacity

**DOI:** 10.3390/toxins13100729

**Published:** 2021-10-16

**Authors:** Vasileios Paraskeuas, Eirini Griela, Dimitrios Bouziotis, Konstantinos Fegeros, Gunther Antonissen, Konstantinos C. Mountzouris

**Affiliations:** 1 Laboratory of Nutritional Physiology and Feeding, Department of Animal Science, Agricultural University of Athens, Iera Odos 75, 11855 Athens, Greece; v.paraskeuas@gmail.com (V.P.); eirinigkr21@gmail.com (E.G.); dbouziotis@hotmail.com (D.B.); cfeg@aua.gr (K.F.); 2 Department of Pathobiology, Pharmacology and Zoological Medicine, Faculty of Veterinary Medicine, Ghent University, Salisburylaan 133, 9820 Merelbeke, Belgium; Gunther.Antonissen@ugent.be

**Keywords:** mycotoxins, broilers, gut health, antioxidant response, detoxification, inflammation

## Abstract

Mycotoxins are a crucial problem for poultry production worldwide. Two of the most frequently found mycotoxins in feedstuffs are deoxynivalenol (DON) and fumonisins (FUM) which adversely affect gut health and poultry performance. The current knowledge on DON and FUM effects on broiler responses relevant for gut detoxification, antioxidant capacity, and health is still unclear. The aim of this study was to assess a range of selected molecular intestinal biomarkers for their responsiveness to the maximum allowable European Union dietary levels for DON (5 mg/kg) and FUM (20 mg/kg) in broilers. For the experimental purpose, a challenge diet was formulated, and biomarkers relevant for detoxification, antioxidant response, stress, inflammation, and integrity were profiled across the broiler intestine. The results reveal that DON significantly (*p* < 0.05) induced aryl hydrocarbon receptor (AhR) and cytochrome P450 enzyme (CYP) expression mainly at the duodenum. Moreover, DON and FUM had specific significant (*p* < 0.05) effects on the antioxidant response, stress, inflammation, and integrity depending on the intestinal segment. Consequently, broiler molecular responses to DON and FUM assessed via a powerful palette of biomarkers were shown to be mycotoxin and intestinal site specific. The study findings could be highly relevant for assessing various dietary bioactive components for protection against mycotoxins.

## 1. Introduction

Contamination of cereal grains and their byproducts by mycotoxins is a worldwide problem negatively affecting poultry production [1]. Two *Fusarium* mycotoxins which are among the most toxic and frequent feed contaminants are the trichothecene deoxynivalenol (DON) and fumonisins (FUM). DON is produced as a secondary metabolite by *Fusarium graminearum* and *Fusarium culmorum*, whereas FUM are secondary metabolites which are mainly produced by *Fusarium verticillioides* and *Fusarium proliferatum* [2,3].

The European Union (EU) limitations for DON and FUM in poultry feed are set to 5 and 20 mg/kg, respectively [4]. However, in recent studies, there have been indications that, even at lower concentrations than the EU limits, DON and FUM could cause mycotoxicosis and negatively affect broiler gut health and performance [1,5], while the severity of mycotoxins on broiler performance will depend on the type of mycotoxins involved and level of feed contamination; other factors such as the overall diet, bird genetics, and the rate of mycotoxin absorption will also have to be taken into consideration [6]. In particular, compared to non-genetically developed traditional broiler strains, the high-performance capacity of modern broilers may render them susceptible to even low mycotoxin levels [7]. Moreover, the fast absorption rates of mycotoxins in the gut mean that the intestinal cells are promptly faced with mycotoxins’ deleterious effects that could result in gut barrier impairments and dysfunctions [5].

Mycotoxins’ damaging effects on broiler gut health are largely related to oxidative stress and subsequent inflammation [3,8]. Therefore, a topic of paramount importance is how mycotoxin-related oxidative stress can be addressed promptly and effectively at the gut level. In this sense, responsive molecular biomarkers whose expression can differentiate depending on the mycotoxin and broiler intestinal site are highly warranted. The cellular cytoprotection against oxidative stress is regulated by the aryl hydrocarbon receptor (AhR) and nuclear factor erythroid-derived 2-like 2 (Nrf2) signaling pathways [9]. The AhR pathway is related to the detoxification of xenobiotic compounds, such as mycotoxins, and the Nrf2 pathway is the main modulator of the antioxidant response [10], responsible for the transcription of phase II antioxidant and cytoprotective genes [11,12,13].

Although there are indications that the AhR pathway responds to *Fusarium* mycotoxins in pig [14], rat [15,16], and mouse [17,18] tissues, more studies are clearly required to address DON and FUM effects on the AhR pathway in broilers at the intestinal level [19]. In addition, *Fusarium* mycotoxins have been shown to negatively impact the broiler antioxidant defense system in the liver [3,20,21,22] and intestine [22]. However, DON and FUM effects on the Nrf2 pathway in broilers at the intestinal level are currently not known. In line with the above, although heat shock proteins (HSPs) are among the important cellular responses to stressors [23,24], there are no studies that have investigated *Fusarium* mycotoxin effects on HSPs across the broiler gut.

Oxidative stress is also known to be related to intestinal immune and epithelial dysfunctions [25,26]. The activation of nuclear transcription factor-κB (NF-κB) is triggered by the activation of Toll-like receptors (TLRs) in the intestinal cell surface, and this pathway is responsible for the induction of inflammatory responses [27]. While there are indications that DON and FUM increased the expression of NF-κB pathway-related genes mainly in the broiler jejunum [5,6,28,29,30], still more work involving intestinal profiling for critical relevant genes is required.

The above indications include the effects of DON and FUM on the expression of tight junction (TJ) proteins such as occludin (OCLN), claudins (CLDNs), zonula occludens (ZO), and mucin 2 (MUC2), known to maintain barrier integrity and tightness [31]. In particular, the current findings on the effects of *Fusarium* mycotoxins on the expression of gut barrier elements range from enhancement [28] and no effect [22] to downregulation [30,32] and increased paracellular permeability [20], clearly highlighting the need for more studies.

Finally, despite all the above, the overall diet obviously plays an important role in accelerating or alleviating conditions leading to oxidative stress and deviations from intestinal homeostasis. For example, broiler diets based on cereals with high concentrations of soluble non-starch polysaccharides (NSP) such as wheat and rye are known to negatively impact nutrient digestibility, gut health, and, as a result, broiler growth performance [33,34]. Moreover, DON and FUM are frequently found as contaminants of wheat and rye or their byproducts [1].

The aim of this study was to assess a range of selected intestinal molecular biomarkers for their responsiveness to the DON and FUM maximum allowable EU dietary levels. For the purpose of this study, a challenge diet [35] was formulated, and biomarkers relevant to detoxification, antioxidant response, stress, inflammation, and barrier integrity were profiled across the broiler intestine.

## 2. Results

### 2.1. Growth Performance Responses

Among growth performance responses, Body Weight Gain (BWG) and Feed Intake (FI) differed significantly (*p* < 0.05) between treatments (Table 1). In particular, broilers of the DON or FUM treatment had significantly lower (*p* = 0.002) BWG compared to broilers of the un-supplemented challenge diet (CD) treatment. In addition, FUM inclusion significantly decreased FI (*p* = 0.018) during the whole experiment compared to the CD treatment (Table 1).

### 2.2. Profile of Selected Gene Expression across the Intestine

In the duodenum, the relative expression levels of AhR pathway (AhR1, AhR2, ARNT, P23, XAP2, CYP1A1, CYP1A2, and CYP1B1), Nrf2 pathway (Nrf2, Keap1, CAT, SOD, GPX2, HMOX1, NQO1, and GSTA2), and heat shock response (HSP70 and HSP90) related genes are presented in Table 2. The inclusion of DON significantly upregulated (*p* < 0.05) the relative gene expression of AhR1 (*p* = 0.005), AhR2 (*p* = 0.038), and CYP1B1 (*p* = 0.041) compared to broilers of the un-supplemented CD treatment. Moreover, the DON treatment showed significantly higher values of NQO1 (*p* = 0.008) compared to FUM. In addition, the gene expression levels of HSP90 were significantly (*p* = 0.020) upregulated by DON supplementation compared to FUM addition. On the other hand, FUM significantly (*p* = 0.010) increased HSP70 expression levels in the duodenum compared to the CD treatment (Table 2).

The expression levels of NF-κΒ pathway (TLR2B, TLR4, NF-κΒ, ΙΚKa, and TNFa) and gut barrier integrity (OCLN, ZO1, ZO2, CLDN1, CLDN5, and MUC2) related genes in the duodenum are presented in Table 3. Broilers supplemented with DON showed significantly (*p* = 0.035) higher expression levels of NF-κB compared to those fed the un-supplemented CD treatment. In addition, MUC2 expression in the duodenum was significantly (*p* = 0.019) decreased by DON compared to the control CD treatment. On the other hand, FUM significantly (*p* = 0.005) increased the expression levels of TLR4 compared to the CD treatment. Finally, in the duodenum, broilers supplemented with FUM showed significantly (*p* = 0.035) higher expression levels of NF-κΒ compared to those of the control CD treatment (Table 3).

In the jejunum, DON inclusion significantly (*p* = 0.037) induced the expression levels of CYP1A1 compared to the CD treatment. On the other hand, broilers fed FUM diets showed significantly (*p* = 0.021) lower levels of GSTA2 compared to those in the CD treatment (Table 4).

Moreover, in the jejunum, MUC2 had significantly (*p* = 0.028) lower expression levels in the FUM treatment than in the CD treatment. All the other NF-κΒ pathway- and gut integrity-related genes did not significantly (*p* > 0.05) differ between treatments (Table 5).

In the ileum, DON and FUM effects on the relative expression levels of genes related to the AhR and Nrf2 pathways and heat shock response are presented in Table 6. In particular, DON supplementation significantly increased ARNT (*p* = 0.029) and XAP2 (*p* = 0.018) expression levels compared to the CD treatment. In addition, DON significantly increased Keap1 (*p* = 0.026) and HSP90 (*p* = 0.011) compared to the CD treatment. By contrast, DON significantly lowered GPX2 (*p* = 0.042) expression levels compared to the CD treatment. On the other hand, the expression of CYP1A2 was significantly (*p* = 0.036) increased by FUM compared to the CD treatment. In addition, the FUM treatment showed significantly lower GPX2 (*p* = 0.042) gene expression levels than the CD treatment (Table 6).

The expression levels of genes related to the NF-κΒ pathway and gut barrier integrity in the ileum are shown in Table 7. The expression levels of NF-κΒ were significantly (*p* = 0.018) higher in the DON treatment compared to the FUM treatment. In addition, DON and FUM significantly (*p* = 0.002) downregulated CLDN1 expression levels compared to the CD treatment (Table 7).

In the ceca, the expression levels of AhR and Nrf2 pathway genes and heat shock response proteins are presented in Table 8. DON supplementation significantly (*p* < 0.05) increased the expression levels of Nrf2 (*p* = 0.035) and Keap1 (*p* = 0.020), compared to the FUM and CD treatments, respectively. Furthermore, the expression levels of HSP90 were significantly (*p* = 0.021) higher in the DON treatment than in the CD treatment. On the other hand, FUM significantly (*p* = 0.010) upregulated CYP1A2 relative expression levels compared to CD. Moreover, Keap1 expression levels were significantly (*p* = 0.020) higher in the FUM treatment than in the control CD treatment. In addition, NQO1 expression levels were significantly (*p* = 0.010) decreased in the FUM treatment compared to the CD treatment. Finally, in the ceca, HSP70 was significantly (*p* = 0.011) increased by FUM inclusion (Table 8).

The expression levels of genes related to the NF-κΒ pathway and gut barrier integrity were not significantly (*p* < 0.05) affected by either DON or FUM (Table 9).

## 3. Discussion

Mycotoxins are secondary metabolites from fungi which are commonly found as contaminants in animal feedstuffs. The most important mycotoxins for poultry production are those produced by *Aspergillus*, *Fusarium*, and *Penicillium* fungal species [2]. One of the greatest problems related to mycotoxins for poultry when found even at low concentrations in feed is their negative impact on nutrient metabolism, gut health and integrity, and, as a result, broiler growth performance [2,5]. Things can become worse in birds under various challenges such as coccidiosis [36] and heat stress [37]. In poultry, two of the most frequently found mycotoxins in cereal grains are deoxynivalenol (DON) and fumonisins (FUM), which are produced from *Fusarium* fungi [32].

Due to the high growth genetic potential of modern broilers, several studies have revealed that the presence of DON and FUM, even at concentrations close to the European Union (EU) limits (5 and 20 mg/kg of diet, respectively), could potentially exert [5,32] harmful effects on broiler gut health status and productivity, or not [1,6,32]. For example, previous research has shown that DON or FUM supplemented at concentrations close to or lower than the EU limits in broiler diets negatively affected intestinal health and function and, as a result, broiler growth performance [7,28,34]. Furthermore, DON and FUM in combination, after their supplementation either at or below the EU limits, have been shown to negatively affect gut health and broiler body weight gain [2,5]. In this respect, the results of the present study show that, within a challenge diet, DON and FUM at the allowable EU maximum limits negatively affected broiler performance compared to the non-mycotoxin-supplemented challenge diet by decreasing BWG. Moreover, FUM supplementation reduced overall feed intake. These inconsistencies regarding the effects of DON and FUM on broiler growth performance could probably be attributed to the source of mycotoxin used in the experimentation, broiler genetics, the levels of mycotoxins in the diet, environmental conditions, and diet type [2].

Taken together, there is a need to identify the mechanisms behind the harmful effects of DON and FUM on broiler gut health and hence performance, in order to better understand which metabolic pathways are involved at each intestinal site. For this reason, the present study evaluated a palette of molecular biomarkers related to the most crucial metabolic pathways for intestinal detoxification, antioxidant response, stress, inflammatory response, and barrier integrity across the broiler intestine.

It is known that mycotoxins could cause intestinal toxicity by inducing oxidative stress [8,38]. Controlling oxidative stress is essential for gut health and performance. Intestinal cells can resist oxidative stress via mechanisms which modulate the gene expression of cytoprotective enzymes with detoxifying and antioxidant functions [9,25]. The aryl hydrocarbon receptor (AhR) pathway is related to the detoxification of xenobiotic compounds such as mycotoxins. In addition, AhR is a nuclear transcription factor expressed in tissues that stimulates the expression of genes related to mycotoxin metabolism [9]. Two types of AhRs are commonly found in avian species, AhR1 and AhR2 [39]. When AhRs are in an inactivated form, they bind in a multiprotein complex which consists of heat shock protein 90 (Hsp90), hepatitis B virus X-associated protein (XAP2), and protein p23 [40]. Mycotoxins and other AhR ligands that activate the AhR signaling pathway bind to AhRs and are then transferred to the nucleus [41]. After this binding, the AhR-ARNT complex binds to xenobiotic responsive elements (XREs) and modulates the expression of xenobiotic-metabolizing enzymes (XMEs) such as quinone oxidoreductase 1 (NQO1), glutathione transferase A2 (GSTA2), and cytochrome P450 (CYP) enzymes (CYP1A1, CYP1A2, CYP1B1) known as phase I enzymes. Moreover, CYP enzymes participate in the oxidative metabolism and elimination of many xenobiotics [9]. In broilers, the effects of mycotoxins on AhR signaling pathway-related genes have been investigated by a limited number of studies. In particular, AhR and CYP enzyme expression levels were increased by the ingestion of aflatoxin [11,42] and T-2 toxin [43] in the chicken liver. However, there is scarce information regarding the effects of DON and FUM on AhR metabolic pathway-related genes across the broiler intestine [19]. In this work, DON mainly affected intestinal detoxification mechanisms at the broiler duodenum and ileum. In particular, the expression levels of AhR1, AhR2, and the CYP1B1 enzyme in the duodenum and ARNT and XAP2 in the ileum were increased by DON ingestion. In addition, upregulation of CYP1A1 expression levels was evidenced by DON in the jejunum. On the other hand, compared to DON, FUM had a lesser and different effect on AhR pathway-related genes by upregulating CYP1A2 expression in the ileum and ceca.

Nuclear factor erythroid-derived 2-like 2 (Nrf2) is a key regulator of the antioxidant response and xenobiotic metabolism in broiler intestinal cells [10,44]. In its inactivated form, Nrf2 is found in the cell cytoplasm bound with its inhibitor Kelch-like ECH-associated protein-1 (Keap1) [45]. Disruption of the Nrf2 and Keap1 complex by ROS leads to Nrf2 translocation to the nucleus where it binds to antioxidant response element (ARE) and drives the transcription of several cytoprotective genes known as phase II enzymes [46]. Phase II proteins, antioxidant enzymes, and detoxifying enzymes such as catalase (CAT), superoxide dismutase (SOD), glutathione reductase (GSR), glutathione peroxidase 2 (GPx2), glutathione S-transferase alpha 2 (GSTA2), NAD(P)H: quinone oxidoreductase 1 (NQO1), and heme oxygenase-1 (HMOX-1) are responsible for preventing oxidative stress and increasing toxin metabolism [11,47]. Previous studies have biochemically shown that feeding low levels of *Fusarium* mycotoxins (DON and/or FUM) close to the EU limits increased lipid peroxidation [3,20,22,23], decreased antioxidant enzyme activity [3,22,23], and, as a result, compromised broilers’ antioxidant capacity. In our study, using a molecular approach, DON and FUM downregulated the antioxidant response in the intestine. In particular, DON’s presence at the EU limit increased Keap1 (the inhibitor of Nrf2) at the ileal and cecal levels and decreased the gene expression of antioxidant enzyme GPX2. Furthermore, FUM supplementation decreased the gene expression of antioxidant enzymes GSTA2 in the jejunum, GPX2 in the ileum, and NQO1 in the ceca and, in addition, upregulated Keap1 in the ceca. On the contrary, a combination of DON (supplemented at 4.96, 12.38, and 24.86 mg/kg) and T-2 toxin (supplemented at 0.23, 1.21, and 2.42 mg/kg) elevated the gene expression of glutathione peroxidase 4 (GPX4) in a dose-dependent manner, showing a continuous increase in the highest supplemented doses [48]. It seems, therefore, that DON and FUM effects on the antioxidant response are related to their inclusion levels in broiler diets. Moreover, the intestinal site dependance evidenced in this study by DON and FUM effects on Nrf2 pathway-related genes provides a possible explanation for the genes’ expression differences which are related with the antioxidant capacity of each intestinal segment induced by *Fusarium* mycotoxins [22].

Heat shock proteins (HSPs) such as HSP70 and HSP90 are expressed as a response to stress factors such as environmental conditions, toxic effects of xenobiotics, oxidative stress, and inflammation [49]. Moreover, HSPs play a key role in the protection and repair of broiler intestinal cells [12]. In the present experiment, DON increased the expression levels of HSP90 in the ileum and the ceca. In accordance with these findings, FUM inclusion increased HSP70 expression levels in the jejunum and ceca, and *Fusarium* mycotoxins increased HSP70 in splenic [24] and liver [23] tissues of 42-day-old broilers. Therefore, in this work, DON and FUM upregulated HSPs, and this could possibly indicate that even at the EU limits, these mycotoxins act as stressors on the intestinal cells. However, to the best of our knowledge, there are no other reports on the effects of *Fusarium* mycotoxins on HSP70 and HSP90 expression levels across the broiler intestine to enable further discussion at this stage.

In this study, in order to evaluate the effects of DON and FUM on the inflammatory response, the expression levels of nuclear factor kappa B (NF-κB) pathway-related genes were measured. In chickens, the activation of Toll-like receptors (TLRs) such as TLR2 and TLR4 leads to the nuclear translocation of NF-κB that controls the expression of pro- and anti-inflammatory genes and hence the inflammatory response [27]. Moreover, this work investigated the effects of DON and FUM on the expression levels of inhibitor of nuclear factor kappa B kinase subunit alpha (IKKa), which is a primary regulator of the NF-κΒ pathway and tumor necrosis factor alpha (TNFa), which plays a key role in the modulation of other inflammatory genes [50]. The study results reveal that DON and FUM induced an inflammatory response mainly in the duodenum. In particular, DON elevated the expression of NF-κB, and FUM increased the expression levels of both NF-κΒ and TLR4 in the duodenum. In accordance with these findings, several studies have shown that dietary *Fusarium* mycotoxins included at concentrations close to EU limits upregulated TLRs [28,51] and pro-inflammatory and anti-inflammatory cytokines [5,6,28,51] in broiler proximal intestinal sites (duodenum and jejunum). However, in the study of [28], increasing the DON concentration up to 5 mg/kg of diet increased TLR and pro-inflammatory cytokine expression levels in the duodenum but downregulated them in the jejunum. On the other hand, DON did not affect TLR and pro-inflammatory cytokine expression levels when supplemented at 10 mg/kg of diet in broilers. It therefore appears that DON and FUM effects on the inflammatory response show dose and intestinal site dependance that merits further investigation.

*Fusarium* mycotoxins and their metabolites act as inhibitors of nucleic acid protein synthesis and, as a result, have a negative impact on intestinal epithelial and immune cells, which are characterized by a high protein turnover [28]. The intestinal epithelial layer is a selective barrier that allows the entrance of dietary nutrients, electrolytes, and water and prevents harmful substances from passing from the intestinal surface to the organism [32]. The gut barrier consists of epithelial cells and tight junction proteins (TJs) which maintain barrier integrity and tightness [31]. TJs include the peripheral membrane protein ZO-1, the transmembrane protein occludin (OCLN), and claudins (CLDNs) [22]. Moreover, mucin 2 (MUC2) is a crucial component of the intestinal mucosa layer, which covers its surface and repairs disruptions caused by factors such as xenobiotics and their metabolites [51]. As a result, downregulation of the expression of TJs and MUC2 might cause impairments to the intestinal barrier and hence reduce nutrient absorption [22]. In the present study, in the duodenum, DON downregulated the expression levels of MUC2, and it decreased the expression levels of CLDN1 in the ileum. Similarly, *Fusarium* mycotoxins consumed at dietary levels close to EU limits decreased the expression levels of MUC2 [22,30] and ZO1 [22] in the jejunum of 42-day-old broilers. Furthermore, FUM decreased MUC2 in the jejunum and CLDN1 in the ileum. Generally, it appears that DON and FUM levels at EU limits have limited effects on TJs, as evidenced mainly in the ileum (CLDN1), while the downregulation of MUC2 occurs more proximally for DON. These findings might be related to the different absorption rates of mycotoxins across the broiler intestine [52]; however, this merits a dedicated investigation.

In the present study, challenge basal diets (CD) were formulated in order to introduce a systemic stressor throughout the experiment. In particular, feeding diets with increased levels of NSP, without the use of NSP enzymes, is known to negatively impact broiler growth performance, nutrient digestibility, and gut function and ecology [33,34,53]. Furthermore, the CD diets were based on wheat and characterized by the inclusion of an additional number of feedstuffs such as sunflower meal, rye, and rapeseed meal with high levels of NSP. The study results confirm the negative impact of the CD diets on performance. In particular, broiler BWG, FI, and FCR were worsened by the CD diets, by 30%, 22%, and 11%, respectively, compared to the performance objectives of 39-day-old male Ross 308 broilers.

**Figure 1:**
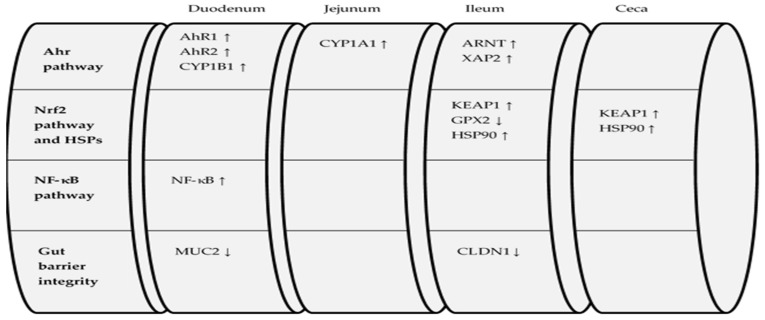
Effects of DON treatment compared to CD treatment on AhR pathway-, Nrf2 pathway-, NF-κB pathway-, and gut barrier integrity-related genes throughout the intestine.

**Figure 2:**
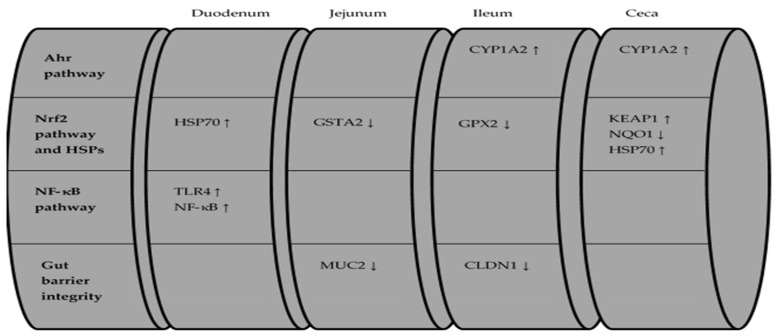
Effects of FUM treatment compared to CD treatment on AhR pathway-, Nrf2 pathway-, NF-κB pathway-, and gut barrier integrity-related genes throughout the intestine.

Overall, the effects of DON and FUM administration on the expression levels of the examined biomarkers are summarized in Figure 1 and Figure 2.

## 4. Conclusions

In conclusion, a powerful palette of molecular biomarkers critical for intestinal detoxification, antioxidant response, stress, inflammatory response, and barrier integrity enabled the detection of the broiler biological responsiveness to DON and FUM, using a dietary challenge model. In particular, the presence of DON and FUM at the maximum allowable European Union (EU) limits upregulated components of the intestinal detoxification process of the AhR pathway. Of the two mycotoxins studied, DON had a stronger and more proximal effect compared to FUM. However, both DON and FUM downregulated components of the Nrf2 antioxidant response pathway across the intestine. The latter could have various shortcomings for broiler antioxidant capacity and control of inflammation. Moreover, DON’s and FUM’s triggering effects on HSPs and components of inflammation (e.g., NF-κB) highlight and confirm the need for adequate mycotoxin control. In this respect, dietary strategies to control oxidative stress and inflammation in broilers need to consider the mycotoxin-relevant and intestinal site-specific effects seen on a series of molecular biomarkers such as the ones assessed in this work. Finally, the knowledge generated at molecular level in this work may also provide useful tools for assessing various bioactive components such as mycotoxin deactivators, yeast cell wall extracts, phytogenics, and probiotics for protection against *Fusarium* mycotoxins.

## 5. Materials and Methods

### 5.1. Animals and Experimental Treatments

A total of 378 1-day-old male Ross 308 broilers were obtained from a commercial hatchery where they were vaccinated against Marek’s disease, infectious bronchitis, and Newcastle disease. Birds were randomly allocated to 3 experimental treatments for 39 days. Each treatment had 7 replicate cages of 18 broilers each. A three-phase feeding program with starter (1 to 13 days), grower (14 to 26 days), and finisher (27 to 39 days) challenge diets was followed (Table 10). The composition of the challenge basal diets in each growing phase was based on wheat and soybean meal with inclusions of sunflower meal, rye, and rapeseed meal (Table 10). Moreover, the basal diets were designed according to [35] in order to act as an additional stressor factor and were not supplemented with non-starch polysaccharide (NSP) enzymes.

The 3 experimental treatments were: CD (challenge diet) without other additions used as control, CD with addition of DON (5 mg/kg of diet), and CD with addition of FUM (20 mg/kg of diet). DON and FUM were added to the CD in order to achieve the maximum allowable concentration limits in the European Union (EU) for poultry, i.e., 5 mg DON/kg of diet and 20 mg FUM/kg of diet, respectively [4].

The mycotoxins DON and FUM for this work were commercially produced from *Fusarium graminearum* and *Fusarium verticillioides*, respectively, and subsequently purified and crystallized (2.21 mg DON/g and 13.7 mg fumonisin B1 (FB1) + fumonisin B2 (FB2)/g) (Biopure-Romer Laboratories Diagnostics GmbH, Tulln, Austria). According to the experimental treatments, feeds were mixed with DON or FUM in order to incorporate them at the maximum allowable EU limits. To ensure a homogeneous distribution of the toxins, mycotoxin premixes were prepared by mixing the DON or FB culture materials in milled wheat at a concentration of 0.167 mg DON/g and 0.667 mg FBs/g, respectively. Subsequently, the premixes were incorporated in the final respective diets per treatment (CD took only milled wheat) at an inclusion rate of 3%, and the mycotoxin contamination of all diets was assessed by a validated high-performance liquid chromatography with tandem mass (HPLC-MS/MS) detection technique (AT-SOP, Romerlabs, Tulln, Austria). Samples were taken at three different locations in each batch, subsequently pooled per batch, and analyzed for mycotoxin contamination. The final mycotoxin concentrations in the finished feeds were found to be in accordance with the planned concentrations, e.g., no nivalenol, DON, 3-acetylDON, 15-acetylDON, FB1, or FB2 was found in the starter and grower control CDs, and only a negligible low level of FB1 (54 ± 14 µg/kg) was found in the finisher control CD; DON starter diet = 3771 ± 453 µg DON/kg; DON grower diet = 5400 ± 648 µg DON/kg; DON finisher diet = 3008 ± 361 µg DON/kg; FUM starter diet = 20,002 ± 2002 µg FB1 and 6183 ± 742 µg FB2/kg; FUM grower diet = 14,748 ± 1475 µg FB1 and 5995 ± 720 µg FB2/kg; and FUM finisher diet = 8134 ± 976 µg FB1 and 5959 ± 715 µg FB2/kg.

The broilers had access to feed and water ad libitum, and the experiment lasted 39 days. The lighting program was light/dark (18 h:6 h). The experimental protocol was in accordance with the EU Directive for the protection of animals used for scientific purposes [54,55] and approved by the relevant national authority (Department of Agriculture and Veterinary Policy, General Directorate of Agriculture, Economy, Veterinary and Fisheries). Broilers were euthanized via electrical stunning prior to slaughter.

### 5.2. Growth Performance Responses

Growth performance responses such as broiler body weight gain (BWG), feed intake (FI), and feed conversion ratio (FCR) were evaluated for the entire duration of the experiment (39 days). The calculation of FCR was conducted according to the following equation: g FI/g BWG.

### 5.3. Organ Sampling

At 39 days of age, 7 broilers per treatment were randomly selected, and small fragments (approximately 5 cm) of the mid-duodenum, mid-jejunum, mid-ileum, and mid-ceca samples were excised carefully and subsequently stored in RNAlater; after a short stay at 4 °C, they were then stored at −80 °C.

### 5.4. Molecular Analyses

The segments without the digesta were washed completely in 4 mL cold phosphate-buffered saline (PBS)-ethylene diamine tetra-acetic acid (EDTA; 10 mmol/L) solution (pH = 7.2), and a small piece (70–100 mg) was transferred to a sterile Eppendorf-type tube. Eventually, the total RNA from the duodenal, jejunal, ileal, and cecal segments was obtained as reported by the manufacturer protocol from Macherey-Nagel GmbH & Co. KG, Dueren, Germany, by handling NucleoZOL Reagent. RNA quantity and quality were verified by spectrophotometry with the use of a NanoDrop-1000 by Thermo Fisher Scientific, Waltham, United Kingdom.

DNAse treatment was applied in order to remove the contaminating genomic DNA from the RNA samples. An amount of 10 μg of RNA was diluted with 1 U of DNase I (M0303, New England Biolabs Inc, Ipswich, UK) and 10 μL of 10 × DNAse buffer to a final volume of 100 µL upon the addition of diethylpyrocarbonate (DEPC) treated water, for 20 min at 37 °C. Before DNAse inactivation at 75 °C for 10 min, EDTA needed to be added to a final concentration of 5 mM to protect RNA from being degraded during enzyme inactivation. RNA integrity was examined by agarose gel electrophoresis.

From each sample, 500 ng of total RNA was reverse transcribed to cDNA by PrimeScript RT Reagent Kit (Perfect Real Time, Takara Bio Inc., Shiga-Ken, Japan) according to the manufacturer’s recommendations. All cDNAs were afterwards stored at –20 °C.

The following *Gallus gallus* genes were examined: aryl hydrocarbon receptor 1 (*Ahr1*), aryl hydrocarbon receptor 2 (*Ahr2*), aryl hydrocarbon receptor nuclear translocator (*ARNT*), prostaglandin E synthase 3 (*P23*), AH receptor-interacting protein (*XAP2*), cytochrome P450 1A1 (*CYP1A1*), cytochrome P450 1A2 (*CYP1A2*), cytochrome P450 1B1 (*CYP1B1*), nuclear factor erythroid-derived 2-like 2 (*Nrf2*), kelch like ECH associated protein 1 (*Keap1*), catalase (*CAT*), superoxide dismutase 1 (*SOD1*), glutathione peroxidase 2 (*GPX2*), heme oxygenase 1 (*HMOX1*), NAD(P)H quinone dehydrogenase 1 (*NQO1*), glutathione S-transferase alpha 2 (*GSTA2*), CREB binding protein (CBP), heat shock protein 70 (*HSP70*), heat shock protein 90 (*HSP90*), Toll-like receptor 2B (*TLR2B*), Toll-like receptor 4 (*TLR4*), nuclear factor κB subunit 1 (*NFKB1*), inhibitor of nuclear factor kappa-B kinase subunit alpha (*IKKa*), tumor necrosis factor alpha (*TNFa*), occludin (*OCLN*), zonula occludens-1 (*ZO1*), zonula occludens-2 (*ZO2*), claudin-1 (*CLDN1*), claudin-5 (*CLDN5*), mucin-2 (*MUC2*), glyceraldehyde 3-phosphate dehydrogenase (*GAPDH*), and actin beta (*ACTB*). Suitable primers were designed using the GenBank (https://www.ncbi.nlm.nih.gov/nuccore/, accessed on 29 April 2020) sequences deposited in the National Center for Biotechnology Information and US National Library of Medicine (NCBI), which are shown in Table 11. Primers were checked using the Primer Blast algorithm (https://www.ncbi.nlm.nih.gov/tools/primer-blast/index.cgi, accessed on 29 April 2020) for *Gallus gallus* mRNA databases to ensure that there was a unique amplicon. The calculation of relative expression ratios of target genes was conducted according to [56] and was adapted for the multi-reference gene normalization procedure using the geometric mean of the linear relative quantity (RQ) values of the 2 reference genes, GAPDH and ACTB, used in the present study, according to [57].

### 5.5. Statistical Analysis

Experimental data were checked for normality using the Kolmogorov–Smirnov test and found to be normally distributed. All data were analyzed with the one-way ANOVA procedure using the SPSS (PASW Statistics 18, SPSS Inc., Chicago, IL, USA) for Windows statistical package program. Statistically significant effects were further analyzed, and the comparison of means was conducted by Tukey’s honestly significant difference multiple comparison procedure. Statistical significance was determined at *p* < 0.05.

## Figures and Tables

**Table 1:** Broiler overall (1–39 days) growth performance responses.

Item ^1^	Treatments ^2^			Statistics	
	CD	DON	FUM	SEM ^3^	*p*-Value ^4^
BWG (g)	1965.6 ^a^	1827.1 ^b^	1816.7 ^b^	38.14	0.002
FI (g)	3387.2 ^a^	3351.8 ^a,b^	3183.9 ^b^	68.06	0.018
FCR (g FI/g BWG)	1.73	1.83	1.75	0.042	0.054

^1^ Body weight gain (BWG), feed intake (FI), and feed conversion ratio (FCR). Data represent means from n = 7 replicate cages of 18 broilers each analyzed per treatment. ^2^ Challenge diet (CD) with no mycotoxin inclusion used as control, CD with deoxynivalenol (DON) addition at 5 mg/kg of diet, and, CD with fumonisin (FUM) addition at 20 mg/kg of diet. ^3^ Pooled standard error of means. ^4^ Within the same row, means with no common superscript per treatment (a, b) differ significantly (*p* < 0.05).

**Table 2:** Relative gene expression of aryl hydrocarbon receptor (AhR) pathway-related genes (AhR1, AhR2, ARNT, P23, XAP2, CYP1A1, CYP1A2, CYP1B1, NQO1, GSTA2), Nrf2 pathway genes (Nrf2, Keap1, CAT, SOD, GPX2, HMOX1), and heat shock proteins (HSP70, HSP90) in the duodenum of 39-day-old broilers.

Duodenum	Treatments ^1^			Statistics	
	CD	DON	FUM	SEM ^2^	*p*-Value ^3^
**AhR Pathway**					
AhR1	0.66 ^b^	2.91 ^a^	1.21 ^a,b^	0.478	0.005
AhR2	1.14 ^b^	2.33 ^a^	1.27 ^a,b^	0.463	0.038
ARNT	1.48	2.29	1.91	0.679	0.498
P23	1.09	2.05	1.64	0.586	0.286
XAP2	0.83	1.70	1.18	0.537	0.287
CYP1A1	1.58	1.60	2.53	0.737	0.358
CYP1A2	1.06	1.63	1.05	0.471	0.390
CYP1B1	1.08 ^b^	2.21 ^a^	1.28 ^a,b^	0.436	0.041
**Nrf2 Pathway**					
Nrf2	1.02	0.98	1.04	0.201	0.956
Keap1	0.92	1.24	1.64	0.323	0.097
CAT	1.34	1.14	0.96	0.229	0.269
SOD	1.08	1.37	1.01	0.200	0.198
GPX2	1.15	1.12	1.06	0.243	0.939
HMOX1	1.15	1.02	0.95	0.212	0.633
NQO1	1.74 ^a,b^	1.90 ^a^	0.89 ^b^	0.341	0.018
GSTA2	1.01	1.49	0.64	0.349	0.078
**Heat Shock Proteins**					
HSP70	0.74 ^b^	1.28 ^a,b^	1.99 ^a^	0.362	0.010
HSP90	1.50 ^a,b^	3.04 ^a^	1.07 ^b^	0.493	0.020

^1^ Challenge diet (CD) with no mycotoxin inclusion used as control, CD with deoxynivalenol (DON) addition at 5 mg/kg of diet, and CD with fumonisin (FUM) addition at 20 mg/kg of diet. Data represent means from n = 7 broilers analyzed per treatment. ^2^ Pooled standard error of means. ^3^ Within the same row, means with no common superscript per treatment (a, b) differ significantly (*p* < 0.05).

**Table 3:** Relative gene expression of NF-κΒ pathway (TLR2B, TLR4, NF-κB, IKKa, TNFa) and gut barrier integrity genes (OCLN, ZO1, ZO2, CLDN1, CLDN5, MUC2) in the duodenum of 39-day-old broilers.

Duodenum	Treatments ^1^			Statistics	
	CD	DON	FUM	SEM ^2^	*p*-Value ^3^
**NF-κΒ Pathway**					
TLR2B	1.33	2.07	1.62	0.674	0.547
TLR4	0.68 ^b^	1.54 ^a,b^	1.72 ^a^	0.548	0.005
NF-κB	0.84 ^b^	1.10 ^a^	1.10 ^a^	0.107	0.035
IKKa	1.58	1.83	1.06	0.245	0.143
TNFa	1.67	1.90	1.27	0.519	0.490
**Gut Barrier Integrity**					
OCLN	1.04	1.07	1.08	0.181	0.975
ZO1	0.95	0.90	1.04	0.140	0.589
ZO2	0.93	1.01	0.99	0.077	0.586
CLDN1	1.09	0.79	0.91	0.193	0.303
CLDN5	0.97	0.76	1.02	0.248	0.539
MUC2	1.68 ^a^	0.93 ^b^	1.02 ^a,b^	0.330	0.019

^1^ Challenge diet (CD) with no mycotoxin inclusion used as control, CD with deoxynivalenol (DON) addition at 5 mg/kg of diet, and, CD with fumonisin (FUM) addition at 20 mg/kg of diet. Data represent means from n = 7 broilers analyzed per treatment. ^2^ Pooled standard error of means. ^3^ Within the same row, means with no common superscript per treatment (a, b) differ significantly (*p* < 0.05).

**Table 4:** Relative gene expression of aryl hydrocarbon receptor (AhR) pathway-related genes (AhR1, AhR2, ARNT, P23, XAP2, CYP1A1, CYP1A2, CYP1B1, NQO1, GSTA2), Nrf2 pathway genes (Nrf2, Keap1, CAT, SOD, GPX2, HMOX1), and heat shock proteins (HSP70, HSP90) in the jejunum of 39-day-old broilers.

Jejunum	Treatments ^1^			Statistics	
	CD	DON	FUM	SEM ^2^	*p*-Value ^3^
**Ahr Pathway**					
AhR1	1.40	2.31	1.45	0.809	0.466
AhR2	1.41	1.61	1.27	0.395	0.696
ARNT	1.73	2.76	2.15	0.755	0.408
P23	1.21	2.54	1.68	0.782	0.253
XAP2	0.86	0.91	1.74	0.480	0.363
CYP1A1	0.71 ^b^	3.23 ^a^	1.71 ^a,b^	0.895	0.037
CYP1A2	1.25	4.48	1.31	1.076	0.086
CYP1B1	1.41	2.88	1.32	0.908	0.186
**Nrf2 Pathway**					
Nrf2	1.07	0.89	0.90	0.232	0.687
Keap1	0.70	1.48	1.68	0.420	0.073
CAT	1.07	1.38	1.03	0.352	0.695
SOD	0.88	1.15	0.86	0.188	0.252
GPX2	1.46	1.19	0.74	0.325	0.110
HMOX1	0.97	1.24	0.99	0.291	0.599
NQO1	1.76	1.71	0.82	0.479	0.117
GSTA2	1.96 ^a^	1.77 ^a,b^	0.62 ^b^	0.470	0.021
**Heat Shock Proteins**					
HSP70	0.77	1.41	1.46	0.401	0.189
HSP90	1.48	2.57	1.18	0.879	0.276

^1^ Challenge diet (CD) with no mycotoxin inclusion used as control, CD with deoxynivalenol (DON) addition at 5 mg/kg of diet, and, CD with fumonisin (FUM) addition at 20 mg/kg of diet. Data represent means from n = 7 broilers analyzed per treatment. ^2^ Pooled standard error of means. ^3^ Within the same row, means with no common superscript per treatment (a, b) differ significantly (*p* < 0.05).

**Table 5:** Relative gene expression of NFκΒ pathway (TLR2B, TLR4, NF-κB, IKKa, TNFa) and gut barrier integrity genes (OCLN, ZO1, ZO2, CLDN1, CLDN5, MUC2) in the jejunum of 39-day-old broilers.

Jejunum	Treatments ^1^			Statistics	
	CD	DON	FUM	SEM ^2^	*p*-Value ^3^
**NF-κB Pathway**					
TLR2B	0.92	1.29	1.49	0.637	0.667
TLR4	1.20	1.37	1.28	0.472	0.939
NF-κB	0.76	1.27	1.65	0.366	0.079
IKKa	1.49	1.89	1.38	0.590	0.665
TNFa	1.58	2.55	1.40	0.721	0.258
**Gut Barrier Integrity**					
OCLN	0.96	0.99	1.04	0.184	0.915
ZO1	1.04	1.02	0.92	0.157	0.737
ZO2	1.02	1.10	0.94	0.204	0.742
CLDN1	1.08	0.92	0.88	0.204	0.592
CLDN5	1.33	1.04	0.95	0.190	0.131
MUC2	1.96 ^a^	0.94 ^a,b^	0.83 ^b^	0.382	0.028

^1^ Challenge diet (CD) with no mycotoxin inclusion used as control, CD with deoxynivalenol (DON) addition at 5 mg/kg of diet, and CD with fumonisin (FUM) addition at 20 mg/kg of diet. Data represent means from n = 7 broilers analyzed per treatment. ^2^ Pooled standard error of means. ^3^ Within the same row, means with no common superscript per treatment (a, b) differ significantly (*p* < 0.05).

**Table 6:** Relative gene expression of aryl hydrocarbon receptor (AhR) pathway-related genes (AhR1, AhR2, ARNT, P23, XAP2, CYP1A1, CYP1A2, CYP1B1, NQO1, GSTA2), Nrf2 pathway genes (Nrf2, Keap1, CAT, SOD, GPX2, HMOX1), and heat shock proteins (HSP70, HSP90) in the ileum of 39-day-old broilers.

Ileum	Treatments ^1^			Statistics	
	CD	DON	FUM	SEM ^2^	*p*-Value ^3^
**AhR Pathway**					
AhR1	1.33	1.48	0.82	0.492	0.387
AhR2	1.67	1.79	1.29	0.647	0.731
ARNT	0.92 ^b^	2.67 ^a^	1.37 ^a,b^	0.619	0.029
P23	1.02	1.80	1.36	0.355	0.071
XAP2	0.77 ^b^	2.35 ^a^	1.20 ^a,b^	0.399	0.018
CYP1A1	0.76	1.43	1.24	0.388	0.229
CYP1A2	0.87 ^b^	1.20 ^ab^	1.83 ^a^	0.342	0.036
CYP1B1	1.13	1.68	1.31	0.363	0.327
**Nrf2 Pathway**					
Nrf2	0.87	0.98	0.95	0.190	0.837
Keap1	0.70 ^b^	1.48 ^a^	1.42 ^a,b^	0.289	0.026
CAT	1.28	1.01	0.92	0.216	0.243
SOD	1.17	1.12	0.95	0.207	0.565
GPX2	1.16 ^a^	0.85 ^b^	0.88 ^b^	0.125	0.042
HMOX1	1.03	1.00	1.13	0.161	0.713
NQO1	1.68	1.50	1.03	0.371	0.225
GSTA2	1.85	1.40	1.10	0.437	0.244
**Heat Shock Proteins**					
HSP70	1.11	1.60	1.19	0.502	0.589
HSP90	0.85 ^b^	2.32 ^a^	1.22 ^a,b^	0.451	0.011

^1^ Challenge diet (CD) with no mycotoxin inclusion used as control, CD with deoxynivalenol (DON) addition at 5 mg/kg of diet, and, CD with fumonisin (FUM) addition at 20 mg/kg of diet. Data represent means from n = 7 broilers analyzed per treatment. ^2^ Pooled standard error of means. ^3^ Within the same row, means with no common superscript per treatment (a, b) differ significantly (*p* < 0.05).

**Table 7:** Relative gene expression of NF-κΒ pathway (TLR2B, TLR4, NF-κB, IKKa, TNFa) and gut barrier integrity-related genes (OCLN, ZO1, ZO2, CLDN1, CLDN5, MUC2) in the ileum of 39-day-old broilers.

Ileum	Treatments			Statistics	
	CD	DON	FUM	SEM ^2^	*p*-Value ^3^
**NF-κB Pathway**					
TLR2B	1.16	1.19	1.44	0.410	0.762
TLR4	1.26	1.22	1.20	0.253	0.966
NF-κB	1.08 ^a,b^	1.33 ^a^	0.87 ^b^	0.147	0.018
IKKa	1.34	1.05	1.31	0.396	0.723
TNFa	1.18	0.98	1.73	0.536	0.371
**Gut Barrier Integrity**					
OCLN	1.32	0.79	1.02	0.229	0.097
ZO1	1.19	1.09	0.88	0.140	0.115
ZO2	1.22	1.00	0.76	0.189	0.078
CLDN1	1.65 ^a^	0.72 ^b^	0.57 ^b^	0.192	0.002
CLDN5	0.95	0.95	0.67	0.170	0.188
MUC2	1.17	0.99	1.27	0.292	0.635

^1^ Challenge diet (CD) with no mycotoxin inclusion used as control, CD with deoxynivalenol (DON) addition at 5 mg/kg of diet, and, CD with fumonisin (FUM) addition at 20 mg/kg of diet. Data represent means from n = 7 broilers analyzed per treatment. ^2^ Pooled standard error of means. ^3^ Within the same row, means with no common superscript per treatment (a, b) differ significantly (*p* < 0.05).

**Table 8:** Relative gene expression of aryl hydrocarbon receptor (AhR) pathway-related genes (AhR1, AhR2, ARNT, P23, XAP2, CYP1A1, CYP1A2, CYP1B1, NQO1, GSTA2), Nrf2 pathway genes (Nrf2, Keap1, CAT, SOD, GPX2, HMOX1), and heat shock proteins (HSP70, HSP90) in the ceca of 39-day-old broilers.

Ceca	Treatments ^1^			Statistics	
	CD	DON	FUM	SEM ^2^	*p*-Value ^3^
**Ahr Pathway**					
AhR1	1.65	2.65	1.46	0.786	0.291
AhR2	1.38	1.91	1.02	0.489	0.214
ARNT	1.92	3.71	2.27	0.941	0.160
P23	1.23	1.51	1.01	0.230	0.122
XAP2	1.05	1.84	1.14	0.375	0.099
CYP1A1	1.25	1.68	0.93	0.562	0.425
CYP1A2	0.89 ^b^	1.31 ^a,b^	2.09 ^a^	0.355	0.010
CYP1B1	1.18	1.60	1.16	0.391	0.461
**Nrf2 Pathway**					
Nrf2	0.96 ^a,b^	1.26 ^a^	0.63 ^b^	0.221	0.035
Keap1	0.85 ^b^	1.53 ^a^	1.56 ^a^	0.255	0.020
CAT	1.19	0.99	0.72	0.265	0.230
SOD	1.08	1.09	1.00	0.210	0.898
GPX2	0.94	1.11	1.15	0.248	0.685
HMOX1	1.11	0.94	0.93	0.178	0.516
NQO1	1.65 ^a^	1.33 ^a,b^	0.69 ^b^	0.347	0.038
GSTA2	1.72	1.15	0.81	0.386	0.084
**Heat Shock Proteins**					
HSP70	0.78 ^b^	0.99 ^a,b^	1.29 ^a^	0.151	0.011
HSP90	1.08 ^b^	2.45 ^a^	1.18 ^b^	0.494	0.021

^1^ Challenge diet (CD) with no mycotoxin inclusion used as control, CD with deoxynivalenol (DON) addition at 5 mg/kg of diet, and, CD with fumonisin (FUM) addition at 20 mg/kg of diet. Data represent means from n = 7 broilers analyzed per treatment. ^2^ Pooled standard error of means. ^3^ Within the same row, means with no common superscript per treatment (a, b) differ significantly (*p* < 0.05).

**Table 9:** Relative gene expression of NF-κΒ pathway (TLR2B, TLR4, NF-κB, IKKa, TNFa) and gut barrier integrity genes (OCLN, ZO1, ZO2, CLDN1, CLDN5, MUC2) in the ceca of 39-day-old broilers.

Ceca	Treatments ^1^			Statistics	
	CD	DON	FUM	SEM ^2^	*p*-Value ^3^
**NF-κB Pathway**					
TLR2B	1.45	1.15	1.65	0.496	0.607
TLR4	0.84	1.22	1.31	0.283	0.064
NF-κB	0.88	1.16	1.07	0.142	0.152
IKKa	1.03	2.27	1.18	0.612	0.112
TNFa	1.91	1.74	1.14	0.610	0.434
**Gut Barrier Integrity**					
OCLN	1.41	1.29	0.66	0.343	0.089
ZO1	0.98	1.13	1.02	0.173	0.673
ZO2	1.32	0.89	1.07	0.271	0.303
CLDN1	1.07	1.13	0.78	0.240	0.325
CLDN5	0.94	0.76	0.92	0.119	0.285
MUC2	0.95	1.31	0.44	0.346	0.065

^1^ Challenge diet (CD) with no mycotoxin inclusion used as control, CD with deoxynivalenol (DON) addition at 5 mg/kg of diet, and, CD with fumonisin (FUM) addition at 20 mg/kg of diet. Data represent means from n = 7 broilers analyzed per treatment. ^2^ Pooled standard error of means. ^3^ Within the same row, means with no common superscript per treatment (a, b) differ significantly (*p* < 0.05).

**Table 10:** Ingredients and nutritional content of basal experimental diets per feeding period (as-fed basis).

Item	Starter (1–13 days)	Grower (14–26 days)	Finisher (26–39 days)
**Ingredients (g/kg)**			
Wheat	419.4	488	510.7
Rye	75	75	75
Soybean meal 48%	242.8	159.9	133.9
Rapeseed scrap	75	100	100
Full-fat soybean meal	70	50	50
Poultry fat	40	50	42.7
Sunflower scrap 28%	25	25	25
Soybean oil	14.9	17.4	30
Monocalcium phosphate	14.3	12	10.8
Limestone	11.6	9.7	8.9
NaCl	2	1.7	1.7
Na-bicarbonate	2.5	3	3
L-Lysine-HCl	1.2	2.2	2.2
DL-Methionine	2	1.4	1.6
L-Threonine	0.3	0.7	0.6
Vitamin premix ^1^	2	2	2
Mineral premix ^2^	2	2	2
**Calculated Chemical Composition (g/kg)**			
Dry matter	889.0	889.8	889.4
AME (Mj/kg)	12.55	12.97	13.18
Crude protein	230	200	190
Crude fat	81.9	91.1	96.3
Crude fiber	39.4	39.6	39.1
dig-Lysine	11.3	10	9.4
dig-TSSA ^3^	8.4	7.2	7.2
dig-Threonine	7.6	6.8	6.3
Calcium	9.6	8.4	7.8
Av. Phosphorus ^4^	4.8	4.2	3.9
Sodium	1.6	1.6	1.6

^1^ Vitamin premix for starter and grower periods (Rovimix 11 Bro Basic, DSM, Netherlands) provided per kg of diet: 3.6 mg retinol (vitamin A), 100 mg cholecalciferol (vitamin D3), 80 mg vitamin E, 9 mg menadione (vitamin K3), 3 mg thiamine, 7 mg riboflavin, 6 mg pyridoxine, 25 mg cyanocobalamin, 50 mg nicotinic acid, 15 mg pantothenic acid, 1.5 mg folic acid, 150 mg biotin. The vitamin premix for the finisher period (Rovimix 12 Bro Basic, DSM, Netherlands) provided per kg of diet: 3.6 mg retinol (vitamin A), 75 mg cholecalciferol (vitamin D3), 50 mg vitamin E, 7 mg menadione (vitamin K3), 3 mg thiamine, 6 mg riboflavin, 6 mg pyridoxine, 25 mg cyanocobalamin, 40 mg nicotinic acid, 12 mg pantothenic acid, 1.2 mg folic acid, 150 mg biotin. ^2^ The mineral premix (Rovimix Bro M, DSM, Netherlands) provided per kg of diet: 400 mg choline chloride, 250 mg Co, 1.5 mg I, 300 mg Se, 50 mg Fe, 130 mg Mn, 20 mg Cu, 100 mg Zn. ^3^ TSAA total sulfur amino acids. ^4^ Available phosphorus.

**Table 11:** Oligonucleotide primers used for gene expression of selected targets by quantitative real-time PCR.

Target	Primer Sequence (5′-3′)	Annealing Temperature (°C)	PCR Product Size (bp)	GenBank (NCBI Reference Sequence)
GAPDH	F: ACTTTGGCATTGTGGAGGGT R: GGACGCTGGGATGATGTTCT	59.5	131	NM_204305.1
ACTB	F: CACAGATCATGTTTGAGACCTT R: CATCACAATACCAGTGGTACG	60	101	NM_205518.1
**AhR Pathway**				
AhR1	F: TTTAGTGTGGCAGGTGGATT R: CCTTGTGCCAATGATGCTATTTG	60	200	NM_204118.2
AhR2	F: TGTGACTGCAGATGGCTACAT R: CAGCTCTGTCGTCCTTGTGG	62	122	NM_001319008.1
ARNT	F: GAGACCAAGGCCCCAACTAC R: TCGGGTGCCTCTTTCTTTCC	62	140	NM_204200.1
P23	F: ACACCAGGAATCGGCAATGT R: GCCTCCACTCCAAATCAGGG	60	87	NM_205398.1
XAP2	F: GTTTATGGGGAGTCAGCGGAA R: TGGGCTCAGTGTGGAGATCA	60	112	NM_204469.1
CYP1A1	F: GTGATGGAGGTGACCATCGG R: ACATTCGTAGCTGAACGCCA	62	165	NM_205147.1
CYP1A2	F: CTGACCGTACACCACGCTT R: CTCGCCTGCACCATCACTTC	62	75	NM_205146.2
CYP1B1	F: CAGTGACTCCGCATCCCAAA R: CCATACGCTTACGGCAGGTT	62	132	XM_015283751.2
**Nrf2 Pathway**				
Nrf2	F: AGACGCTTTCTTCAGGGGTAG R: AAAAACTTCACGCCTTGCCC	60	285	NM_205117.1
Keap1	F: GGTTACGATGGGACGGATCA R: CACGTAGATCTTGCCCTGGT	62	135	XM_025145847.1
CAT	F: ACCAAGTACTGCAAGGCGAA R: TGAGGGTTCCTCTTCTGGCT	60	245	NM_001031215
SOD1	F: AGGGGGTCATCCACTTCC R: CCCATTTGTGTTGTCTCCAA	60	122	NM_205064.1
GPX2	F: GAGCCCAACTTCACCCTGTT R: CTTCAGGTAGGCGAAGACGG	62	75	NM_001277854.1
HMOX1	F: ACACCCGCTATTTGGGAGAC R: GAACTTGGTGGCGTTGGAGA	62	134	NM_205344.1
NQO1	F: GAGCGAAGTTCAGCCCAGT R: ATGGCGTGGTTGAAAGAGGT	60.5	150	NM_001277619.1
GSTA2	F: GCCTGACTTCAGTCCTTGGT R: CCACCGAATTGACTCCATCT	60	138	NM_001001776.1
**Heat Shock Proteins**				
HSP70	F: ATGCTAATGGTATCCTGAACG R: TCCTCTGCTTTGTATTTCTCTG	60	145	NM_001006685.1
HSP90	F: CACGATCGCACTCTGACCAT R: CTGTCACCTTCTCCGCAACA	60	196	NM_001109785.1
**NFκΒ Pathway**				
TLR2B	F: CTTGGAGATCAGAGTTTGGA R: ATTTGGGAATTTGAGTGCTG	62	238	NM_001161650.1
TLR4	F: GTCTCTCCTTCCTTACCTGCTGTTC R: AGGAGGAGAAAGACAGGGTAGGTG	64.5	187	NM_001030693.1
NF-κB1	F: GAAGGAATCGTACCGGGAACA R: CTCAGAGGGCCTTGTGACAGTAA	59	131	NM_205134.1
IKKa	F: TTCACTGGTAAGCTCCAGCC R: TTCTCTTGCCTCCTGCAACA	60	199	NM_001012904.1
TNFa	F: GAGCAGGGCTGACACGGAT R: GCACAAAAGAGCTGATGGCAG	60	149	NM_204267.1
**Gut Barrier Integrity**				
OCLN	F: TCATCGCCTCCATCGTCTAC R: TCTTACTGCGCGTCTTCTGG	62	240	NM_205128.1
ZO1	F: CTTCAGGTGTTTCTCTTCCTCCTC R: CTGTGGTTTCATGGCTGGATC	59.5	131	XM_413773
ZO2	F: CGGCAGCTATCAGACCACTC R: CACAGACCAGCAAGCCTACAG	59.5	87	NM_204918
CLDN1	F: CTGATTGCTTCCAACCAG R: CAGGTCAAACAGAGGTACAAG	59.5	140	NM_001013611
CLDN5	F: CATCACTTCTCCTTCGTCAGC R: GCACAAAGATCTCCCAGGTC	59.5	111	NM_204201
MUC2	F: GCTGATTGTCACTCACGCCTT R: ATCTGCCTGAATCACAGGTGC	62	442	XM_015274015.1

F—forward; R—reverse. GAPDH = glyceraldehyde 3-phosphate dehydrogenase; ACTB = actin, beta; Ahr1 = aryl hydrocarbon receptor 1; Ahr2 = aryl hydrocarbon receptor 2; ARNT = aryl hydrocarbon receptor nuclear translocator; P23 = prostaglandin E synthase 3; XAP2 = AH receptor-interacting protein; CYP1A1 = cytochrome P450 1A1; CYP1A2 = cytochrome P450 1A2; CYP1B1 = cytochrome P450 1B1; Nrf2 = nuclear factor erythroid-derived 2-like 2; Keap1 = kelch like ECH associated protein 1; CAT = catalase; SOD1 = superoxide dismutase 1; GPX2 = glutathione peroxidase 2; HMOX1 = heme oxygenase 1; NQO1 = NAD(P)H quinone dehydrogenase 1; GSTA2 = glutathione S-transferase; HSP70 = heat shock protein 70; HSP90 = heat shock protein 90; TLR2B = Toll-like receptor 2; TLR4 = Toll-like receptor 4; NF-κΒ1 = Nuclear Factor Kappa B Subunit 1; IKKa = inhibitor of nuclear factor kappa-B kinase subunit alpha; TNFa = Tumor necrosis factor alpha; OCLN = occludin; ZO1 = zonula occludens-1; ZO2 = zonula occludens-2; CLDN1 = claudin-1; CLDN5 = claudin-5; MUC2 = mucin-2.

## Data Availability

The data analyzed during the current study are available from the corresponding author on reasonable request.
